# Phenobarbital does not worsen outcomes of neonatal hypoxia on hippocampal LTP on rats

**DOI:** 10.3389/fneur.2023.1295934

**Published:** 2023-11-21

**Authors:** Irene Sanchez-Brualla, Anjik Ghosh, Viktoriya A. Gibatova, Sean Quinlan, Eric Witherspoon, Stefano Vicini, Patrick A. Forcelli

**Affiliations:** ^1^Department of Pharmacology and Physiology, Georgetown University, Washington, DC, United States; ^2^Interdisciplinary Program in Neuroscience, Georgetown University, Washington, DC, United States; ^3^Department of Neuroscience, Georgetown University, Washington, DC, United States

**Keywords:** anticonvulsant, CA1, Schaeffer collaterals, LTP, sex differences, neonatal development

## Abstract

**Introduction:**

Neonatal hypoxia is a common cause of early-life seizures. Both hypoxia-induced seizures (HS), and the drugs used to treat them (e.g., phenobarbital, PB), have been reported to have long-lasting impacts on brain development. For example, in neonatal rodents, HS reduces hippocampal long-term potentiation (LTP), while PB exposure disrupts GABAergic synaptic maturation in the hippocampus. Prior studies have examined the impact of HS and drug treatment separately, but in the clinic, PB is unlikely to be given to neonates without seizures, and neonates with seizures are very likely to receive PB. To address this gap, we assessed the combined and separate impacts of neonatal HS and PB treatment on the development of hippocampal LTP.

**Methods:**

Male and female postnatal day (P)7 rat pups were subjected to graded global hypoxia (or normoxia as a control) and treated with either PB (or vehicle as a control). On P13-14 (P13+) or P29-37 (P29+), we recorded LTP of the Schaffer collaterals into CA1 pyramidal layer in acute hippocampal slices. We compared responses to theta burst stimulation (TBS) and tetanization induction protocols.

**Results:**

Under the TBS induction protocol, female rats showed an LTP impairment caused by HS, which appeared only at P29+. This impairment was delayed compared to male rats. While LTP in HS males was impaired at P13+, it normalized by P29+. Under the tetanization protocol, hypoxia produced larger LTP in males compared to female rats. PB injection, under TBS, did not exacerbate the effects of hypoxia. However, with the tetanization protocol, PB – on the background of HS – compensated for these effects, returning LTP to control levels.

**Discussion:**

These results point to different susceptibility to hypoxia as a function of sex and age, and a non-detrimental effect of PB when administered after hypoxic seizures.

## 1 Introduction

Early-life seizures are a significant cause of morbidity, and are associated with long-term changes in brain function and behavior (1, 2). A common cause of neonatal seizures is hypoxic–ischemic encephalopathy (HIE) (1). Hypoxia/HIE-induced neonatal seizures are associated with impaired brain development, an increased risk of later-in-life seizures, and cognitive disabilities in both patients (1, 2) and rodent models (3–5). Seizures caused by HIE are generally treated with antiseizure medications, of which phenobarbital (PB) is the most frequently used (6). However, PB is also associated with adverse neurodevelopmental consequences after neonatal exposure. These effects include induction of neuronal apoptosis (7, 8), disrupted synaptic maturation (9, 10) in rats, and cognitive impairment in human (11) and animal studies (7–9, 12–14).

Graded global hypoxia is a well-established rodent model, which recapitulates some features of HIE. While this model does not recapitulate the ischemic component of injury, it does produce both acute and chronic seizures, changes in synaptic development, and long-term emergence of seizure activity, in the absence of acute cell death (3). In this model, hippocampal AMPA receptor function and expression are altered (15), which may lead to alterations in long-term potentiation (LTP) (16, 17) – the persistent increase in synaptic transmission that is a likely substrate for learning and memory in the hippocampus (18). Consistent with this, neonatal hypoxia is associated with impaired learning and memory in hippocampal-dependent tasks in rodents [reviewed in (19)].

Neonatal exposure to PB also impacts hippocampal neurophysiology, although its impacts on LTP have not previously been studied. We have previously reported that a single injection of PB on postnatal day (P)7 increases the frequency of miniature inhibitory postsynaptic currents (mIPSC) in CA1 pyramidal neurons at postnatal day (P)14 rats while decreasing their frequency at P29+ (10). Treatment was also associated with a striking persistence of giant depolarizing potentials (GDPs), which are common early in development but are essentially absent by the second postnatal week in normal animals (10).

Thus, both hypoxia-induced seizures and PB pharmacotherapy can alter the normal course of brain development, although little is known about how these two processes interact. In the clinic, the separate effects of seizures and medication cannot be compared because the outcomes are confounded – infants without seizures are not given medication, and infants with seizures are almost universally treated with anti-seizure medications. The one preclinical study that examined the functional impact of PB on the background of hypoxia found a benign profile – PB exposure did not alter hypoxia-induced seizure-associated cell death and did not worsen behavioral outcomes (20). Whether this profile holds true at the synaptic level remains to be determined. For this reason, additional preclinical studies are needed to separate and determine the contribution of each factor.

Thus, to determine the effects of PB in the hippocampus in the presence or absence of HS, we evaluated the effect of these treatments on hippocampal LTP. Given that there appears to be a complex interaction between hypoxia and age, and similarly, between drug exposure and age, we assessed LTP at two distinct postnatal stages (the second and fourth/fifth postnatal weeks). Moreover, given the reports of sex-dependent effects of hypoxia (21–23), and sex differences in hippocampal development (24) and function (21, 22, 25), we separately evaluated the impact in male and female rats.

## 2 Materials and methods

### 2.1 Experimental design

**Figure 1:**
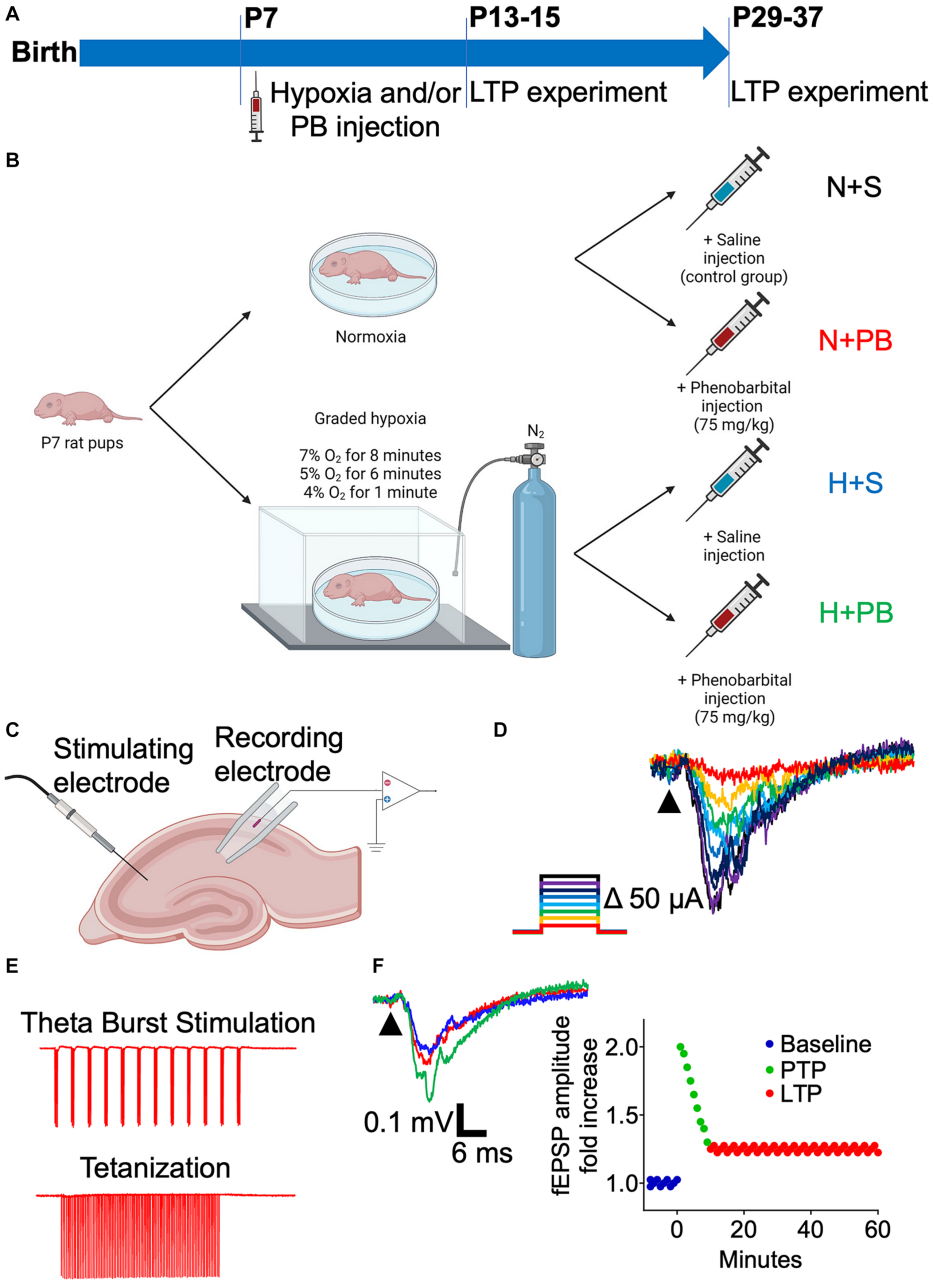
Schematic of the experimental design. **(A)** Timeline of the procedures. **(B)** Schematic of the treatments of the four experimental groups. P7 pups were subjected to a protocol of graded hypoxia or were kept normoxic. Afterwards, they received an i.p. injection of either saline solution or phenobarbital diluted in saline solution (75 mg/kg). **(C)** Schematic illustrating the placement of the stimulating and recording electrodes on hippocampal slices for LTP recordings. **(D)** Representative traces showing superimposed EPSPs resulting from increasing current pulses, on an Input–Output curve color coded for intensity. **(E)** Example of one run of each of the protocols used to induce LTP: Theta Burst Stimulation and tetanization. **(F)** Example traces of the baseline (dark blue), Post-Tetanic Potentiation (PTP, green), and Long-Term Potentiation (LTP, red) on a hippocampal slice. Representation of these three phases on a time vs. fEPSP amplitude fold increase graph.

We used a factorial design to compare the effects of hypoxia and PB and their interaction. On P7, animals were subjected to either graded global hypoxia (H) or normoxia (N; control) and treated with either PB or saline (S) as a vehicle control. We separately compared males and females for each condition. We recorded local field potentials from acute hippocampal slices on P13+ or P29+. We compared the outcomes with a theta burst stimulation protocol to those with a tetanizing stimulation protocol. Our primary outcomes of interest were (1) the impact of hypoxia on LTP, (2) the impact of PB on LTP, and (3) the degree to which PB modified hypoxia-induced LTP changes. Secondary outcomes included (1) sex differences, and (2) developmental time course. For each litter of animals (see below), pups were briefly removed from their dam, weighed, and assigned in a counterbalanced manner to yield similar numbers and similar average weights across the four treatment groups (H + S, H + PB, N + S, N + PB) for each litter. The experimental design is described graphically in Figure 1.

### 2.2 Animals

Timed-pregnant Sprague–Dawley rats were obtained from Envigo (Indianapolis, IN, United States). Upon arrival at Georgetown University, they were housed on a standard 12:12 h light/dark cycle with food and water available *ad libitum*. The date of birth was monitored and designated as P0. Thirty-four independent litters contributed to this study.

For each litter we used for the study, we balanced treatments (N + S, H + S, N + PB, H + PB), and survival interval (P13-15 or P29-37). We weighed all pups at P7 and distributed them in a balanced manner across the 4 treatment groups to ensure similar average body weight at the time of hypoxia and drug injection. Litters ranged between 4 and 16 pups, and thus we were not always able to perfectly balance treatments across sex, treatment, and age at which the experiment was performed. We used two age ranges (P13-15 and P29-37) to balance the desire to make full use of all animals in a litter with the desire to maintain relatively small windows to minimize developmental differences. We have used similar age ranges in the past when examining the impact of PB on hippocampal synaptic development (10). We balanced age within these subgroups, and the age did not systematically vary between treatments (Supplementary Tables S2–S5).

The procedures described in this manuscript were performed with the approval of the Georgetown University Institutional Animal Care and Use Committee (Protocol #2016–1306) and in accordance with the Guide for the Care and Use of Laboratory Animals. The final number of animals (number of litters shown in parenthesis) included in the study are shown in Supplementary Table S1.

### 2.3 Graded global hypoxia

Graded global hypoxia was induced as previously described (15, 26). P7 rat pups were introduced into an airtight chamber (on a heating pad at 32–34°C) connected to an OxyCycler controller (BioSpherix, Ltd. Parish, NY, United States). The OxyCycler controller measured O_2_ concentration and infused 100% N_2_ gas to decrease ambient O_2_ concentration to 7% O_2_ concentration for 8 min, 5% for 6 min, and 4% for 1 min. During hypoxia induction, we monitored all animals for automatisms indicative of seizure activity. Our study endpoints precluded EEG monitoring for all subjects, as this would have required surgical intervention (and thus anesthesia), which would have confounded the outcomes as anesthesia has *per se* effects on brain development (27). However, we have previously used this protocol, and have reported, consistent with the literature, the emergence of spontaneous seizures later in life (28). Moreover, to verify EEG seizure activity, in a subset of animals not used for slice recording, we implanted EEG electrodes-see *EEG recording and analysis* section-and monitored for epileptiform activity during graded hypoxia. We also monitored oxygen saturation in blood simultaneously (SPO2) using a MouseOx Plus pulse oximeter (Starr Life Sciences, RRID:SCR_022984) with a SLS-EA-015021 sensor (Braintree Scientific, Inc.).

While we administered the graded hypoxia protocol to a subset of the rats from each litter, a control cohort from the same litter was placed in an empty cage on a heating pad to control for the brief maternal separation. After the graded hypoxia protocol was completed, all rats were injected with either saline solution or PB (see below).

### 2.4 Drug treatment

Sodium phenobarbital (75 mg/kg; Sigma, St. Louis, MO, United States) was dissolved in 0.9% saline solution at a concentration of 7.5 mg/mL. Following hypoxia (or normoxia in controls), 0.01 mL/g of the drug was injected intraperitoneally, as previously described (10). Phenobarbital (or vehicle) was administered immediately after the completion of the global graded hypoxia protocol for each litter. Control animals received an equivalent volume of normal saline.

This dose of PB (75 mg/kg) was selected based on prior reports of PB efficacy in developing animals and falls at the high end of the anticonvulsant range in neonatal rats (7, 8, 29). This dose was also selected based on its ability to induce neuronal apoptosis (8) and impair hippocampal synaptic maturation (10). The dose used corresponds to the mid-range dose for inducing neuronal cell death (8). A variety of reports have found PB to be effective on a range of doses from 15 to 80 mg/kg (29–32). On the basis of body surface area allometric scaling the dose we used (75 mg/kg) would fall within the human 20–25 mg/kg bolus dose range (33).

We validated dose levels based on pharmacokinetic endpoints. We injected P7 rats with PB (75 mg/kg) and collected trunk blood 1, 4, 16 and 32 h after administration. Samples were collected into lithium heparin coated vials, spun and plasma removed and stored at-80°C until analysis. Plasma levels were measured using a barbiturate ELISA kit (Neogen, 130619). Samples were serially diluted (1:1,000) in EIA buffer to ensure they fell within the dynamic range of the assay. A phenobarbital standard curve was prepared in plasma (0–200 μg/mL), and the assay was performed = according to manufacturer’s instructions.

Following treatment, pups were returned to their home cage with their dam where they were maintained until P13-P15; at that time, some of them were used for LTP experiments. For P29+ recordings, pups were weaned at P21-23 and housed in groups of 2–4 until the day of the experiment (P29-37).

### 2.5 Brain slice preparation

Acute hippocampal slices were prepared as previously described (10). Briefly, P13+ rats were decapitated under isoflurane anesthesia, brains were removed into 4°C sucrose cutting solution. The cutting solution contained (in mM, all from Fisher Scientific): NaCl (87.3), KCl (2.7), CaCl_2_ (0.5), MgSO_4_ (nonhydrate) (6.6), NaH_2_PO_4_ (1.4), NaHCO_3_ (26.0), glucose (25.0), sucrose (75.1) (all from Sigma, St. Louis, MO, United States).

A Vibratome 3000 Plus Sectioning System (Vibratome, St. Louis, MO, United States) was used to prepare transverse slices. The slices were incubated in artificial cerebrospinal fluid (aCSF) containing (in mM): NaCl (123.9), KCl (4.5), Na_2_HPO_4_ (1.2), NaHCO_3_ (26.0), CaCl_2_ (2.0), MgCl_2_ (1.0), and glucose (10.0) at 305 mOsm at 32°C for 30 min. The slices were then incubated for an additional 30 min in the same solution at room temperature. All solutions were continuously bubbled with 95% O_2_/5% CO_2_ to maintain a pH of 7.4.

P29+ rats were anesthetized with isoflurane and perfused with 4°C NMDG (N-methyl-D-glucamine) recovery solution containing (in mM, all from Fisher Scientific): N-methyl-D-glucamine (93), KCl (2.5), NaH_2_PO_4_ (1.2), NaHCO_3_ (30.0), HEPES (20.0), glucose (25.0), ascorbic acid (5.0), thiourea (2.0), sodium pyruvate (3.0), N-acetyl-L-cysteine (5.0), MgSO_4_ (10.0), and CaCl_2_ (0.5) at 305 mOsm and pH 7.4. After perfusion, they were decapitated, their brains were extracted and transverse slices were prepared as described for the younger animals. The slicing took place on ice-cold NMDG solution. Slices were then incubated in a slice chamber, with NMDG solution at 30–32°C for 10 min, and NaCl was added through spiking at the NMDG solution in the chamber with 0.2 mM NaCl in NMDG solution (34).

After the 10 min incubation, the slices were transferred to another slice chamber, filled with HEPES recovery solution: NaCl (92.0), KCl (2.5), NaH_2_PO_4_ (1.2), NaHCO_3_ (30.0), HEPES (20.0), glucose (25.0), ascorbic acid (5.0), thiourea (2.0), sodium pyruvate (3.0), N-acetyl-L-cysteine (5), MgSO_4_ (2), and CaCl_2_ (2) at 305 mOsm and pH 7.4.

After a 4 h incubation, electrophysiological recordings were performed in aCSF as described with the young rats. The solutions containing the slices were constantly bubbled with 95% O_2_/5% CO_2_. Recordings were performed at room temperature (24°C).

### 2.6 LTP recording

Slices were visualized using an upright microscope (Axioscope 2 FS Plus, Zeiss, Germany) equipped with a 2.5x Plan-NEOFLUAR objective and camera. Recording electrodes with a resistance of ~1.0–1.5 MΩ were prepared from borosilicate glass capillaries (Wiretrol II; Drummond, Broomall, PA, United States), pulled using a vertical electrode puller (Narishige, PC-100). All recordings were filtered during acquisition using a 1 kHz high-pass filter and digitized at 20 kHz using a MultiClamp 700B amplifier (Molecular Devices, RRID:SCR_018455).

Electrodes for recording the local field potentials were filled with aCSF and placed in the stratum radiatum of CA1. Stimulation was performed with a bipolar concentric electrode, placed in proximity to the Schaffer collaterals.

We recorded Input–Output curves for the field Excitatory Postsynaptic Potential (fEPSP) and subsequently used a stimulation intensity that produced 30%–50% of the maximum response (Figure 1). Slices were stimulated every 30 s for at least 5 min to obtain a stable baseline. A Theta Burst Stimulation (TBS) or tetanization protocol was then used to induce LTP.

Following the LTP-inducing protocols, we stimulated the slice every 30 s for 1 h to observe the post-tetanic potentiation of the fEPSP and the LTP of this measure. For the TBS protocol (Figure 1E, top), we used 12 trains of four pulses (50 μs) delivered at 100 Hz. Each burst was separated by 200 ms, as described in Abrahamsson et al. (35). We repeated this protocol 3 times, separated by 10 s intervals, for a total of 36 trains of pulses. For the tetanization protocol (Figure 1E, bottom), we used four trains of pulses (100 μs pulse width, 1 s train duration) delivered at 100 Hz. Each train was separated by 10 s (36, 37).

### 2.7 LTP analysis

Data analysis was performed using Clampfit 10 software (Molecular Devices). The amplitude, of the fEPSP was obtained from the I/O curve and the baseline and after the LTP induction protocols. The amplitude of the stimulation artifact was obtained from LTP recordings and its stability was used as a measure of the quality of the slice: recordings from slices that showed significant changes in the stimulus artifact were excluded (see below). Investigators were blind to the treatment that each animal had received during data analysis.

fEPSP amplitudes during LTP were normalized to the mean of the baseline to be able to compare LTP across different slices, and averaged across slices from the same animal. To obtain the value of the post tetanic potentiation (PTP) for the study, we averaged the normalized responses of the first 5 min after we applied the induction protocol, either TBS or tetanization.

In some slices, the average value of the last 30 min of the recording fell below the baseline at the start of the recording session, or recordings displayed drift below 80% of the baseline for ≥ 10 min. This may reflect unstable slice conditions or a stimulating or recording electrode drift and these slices were excluded from further analysis. In a very small number of slices (1.25%), we observed a failure of post-tetanic potentiation and these slices were also excluded.

### 2.8 EEG recording and analysis in neonatal animals

To confirm seizure activity during global hypoxia, we implanted a small group of animals with EEG electrodes. We anesthetized P7 rats with cold anesthesia (placed on wet ice for ~10 min) as previously described (38). A midline scalp incision was placed and three small holes were placed in the skull through which stainless steel recording wire electrodes were placed on top of the dura/brain. The wires were routed to a Pinnacle mouse EEG pedestal (8235-SM). Electrodes were placed over the left and right frontal cortices and the left and right occipital cortex, with a ground/reference placed over the cerebellum.

The implant was secured to the skull with dental acrylic. EEG recordings on newborn rats were performed the same day after at least 1–3 h of recovery. At the time of the recording, the rats were placed into the hypoxia chamber and connected to a headstage/preamplifier which was connected to an amplifier and a data acquisition system. We recorded EEGs while we performed the graded hypoxia protocol on the rats.

### 2.9 EEG recording and analysis in adult animals

To confirm the expected emergence of electrographic seizure activity (spike-and-wave discharges) in adult animals exposed to graded global hypoxia on P7, and to determine if PB exposure altered this outcome, we exposed P7 animals to hypoxia and treated a subset with saline and a subset with phenobarbital. We compared this to a control group that received normoxia and saline. At P60, rats were anesthetized with a combination of dexmedetomidine and ketamine, placed into a stereotaxic frame, and 6 cortical screw electrodes (PlasticsOne, cat: E363/20) were positioned over the left and right frontal cortex, left and right parietal cortex, and ground reference electrodes over the cerebellum. Electrodes were routed into a 6 channels electrode pedestal (PlasticsOne, cat: MS363) and secured to the skull with dental acrylic. EEG recording and analysis followed the procedures we have previously described (28).

Animals were allowed to recover for a minimum of 1 week before recording. At the time of recording animals were placed in a plexiglass monitoring cage and connected to a headstage/preamplifier which was connected to an amplifier and data acquisition system to collect EEG activity. A slip ring commutator was also used to allow for free movement of the animal during the 48 h recording period. Food and water were provided *ad libitum* and animals were maintained on their normal light–dark cycle.

EEGs were analyzed by a reviewer blind to treatment, using Assyst Seizure detection software (Kaoskey, Inc.) with manual review by the trained reviewer. Parameters for detection were customized to detect the spike-and-wave discharges evident in animals exposed to early-life hypoxia. Criteria for spike-and-wave discharges include Criterion for scoring a SWD included: (1) the presence at least five spikes that were > 2x baseline signal; (2) a spike frequency of ∼5–50 Hz; and (3) the presence of the activity on at least two cortical electrode channels.

### 2.10 Statistical analysis

Data were analyzed by mixed effect models or analysis of variance with sex, age, hypoxia exposure, and drug treatment as independent variables.

Input–Output curves were analyzed using GraphPad Prism (Version 8, GraphPad Software, La Jolla, CA, United States, RRID:SCR_002798). Groups were divided by sex and age, and in each of these groups, the four experimental conditions were compared using a three-way ANOVA of repeated measures, using the factors (1) Normoxia-Hypoxia, (2) Drug treatment, (3) Time. Post tetanic potentiation data were also analyzed using GraphPad Prism: groups were divided by sex, age and induction protocol (TBS or tetanization) and in each of these divisions, we compared the four treatment groups using a Kruskal-Wallis test followed by Dunn’s post-test. LTP data were analyzed using IBM® SPSS® Statistics 28.0 (RRID:SCR_016479). We considered the factors (1) Normoxia-Hypoxia, (2) Drug treatment, (3) Sex. We used a mixed model analysis of estimated marginal means, and we established pairwise comparisons between groups that shared all the factors but one. For EEG recordings, data were analyzed using Kruskal-Wallis test.

For all statistical tests, *p*-values <0.05 were considered statistically significant. Familywise error rate was maintained at 0.05 for multiple comparisons. Statistical analyses were performed using SPSS (RRID:SCR_016479) and GraphPad Prism (RRID:SCR_002798). Figures were generated using GraphPad Prism and BioRender (RRID:SCR_018361). Diagrams appearing in the figures were created in BioRender.

## 3 Results

### 3.1 Model validation

**Figure 2:**
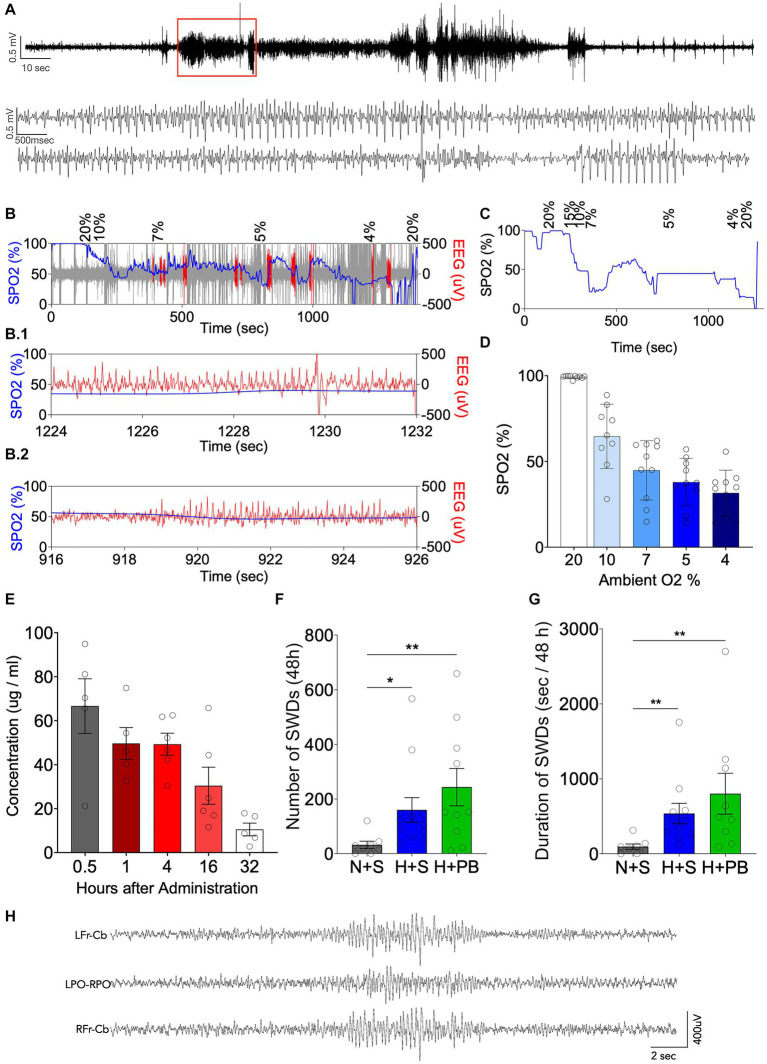
Neonatal graded hypoxia causes seizures and phenobarbital does not reduce neonatal hypoxia-induced epileptogenesis. **(A)** Representative EEG recording from an animal during the induction of hypoxia. Expanded time scale shown in the lower traces. **(B)** Oxygen saturation in blood (SPO2, blue) vs. EEG (μV, red) along time during 15 min of graded hypoxia induction. Expanded time scale shown in B.1 and B.2. **(C)** SPO2 along time during 15 min of graded hypoxia induction. **(D)** Average SPO2 at different oxygen concentrations corresponding to phases of the graded hypoxia protocol. **(E)** Pharmacokinetics of phenobarbital (PB) up to 32 h post administration. **(F)** Number of SWDs over 48 h. **(G)** Total SWD time (s/48 h), measured as the duration in seconds that each rat was seizing over a period of 48 h. **(H)** Representative SWD from a hypoxia exposed animal in the vehicle group. *Significant differences between group means, *p* < 0.05; **Significant differences between group means, *p* < 0.01. Kruskal-Wallis test, Dunn post-test of pairwise comparison between each group mean (H + S, N + PB and H + PB) to the control group mean (N + S). LFr – CB, left frontal lead referenced to cerebellum; LPO-RPO, Left Posterior Parietal referenced to Right Posterior Parietal; RFr – CB, right frontal lead referenced to cerebellum.

We monitored the behavior of all rat pups we subjected to hypoxia at P7 and found automatisms indicative of seizures. Out of a total of 113 rats, 85 showed head bobbing behavior (75% of them), 68 showed jerks or other clonus behavior (60%), and 34 showed wet-dog shakes (30%). Most of the rat pups showed a combination of two or more of these types of behavior. Only 4 rat pups (3.5% out of the total) did not show any semiology that would indicate they were having a seizure. This is consistent with a prior study that observed 93% of pups displaying behavioral and EEG seizures (3). We verified this in a subset of animals by EEG (*n* = 10), as shown in Figure 2. Simultaneous pulse oximetry and EEG recording showed both the expected drop in blood oxygen saturation (Figures 2B–D) and the emergence of epileptiform activity in the EEG (Figures 2A,B). 100% of the implanted rats showed putative epileptiform activity at some point of the graded hypoxia protocol.

To confirm that our phenobarbital dose selection fell within the clinically relevant range, we measured plasma PB levels (Figure 2E) after a single i.p. injection of 75 mg/kg. PB displayed the expected half-life of ~12 h (39). Calculated C_0_ was 58.3 μg/mL, and Tmax was 0.5 h, with a mean plasma level of 66 μg/mL. Plasma levels from 1 to 4 h after administration averaged 49 μg/mL, this fell to 25 μg/mL by 16 h and 10 μg/mL by 32 h. These values are within the range previously reported in immature animals. For example, 60 mg/kg phenobarbital in P19 CD1 mice produced plasma levels of about 40 μg/mL (40), and 75 mg/kg phenobarbital in P7 Wistar rates produced blood concentrations of about 65 μg/mL (7). Moreover, clinical studies have reported plasma concentrations with targets as high as 100 μg/mL (41), particularly for infants with refractory seizures (42). with plasma concentrations in the range of 40–50 μg/mL (43) associated with a plateau in the therapeutic effect (41). This is approximately the same blood concentration of PB that our animals had for at least the first 4 h post injection. Thus, our dose selection falls within the clinically relevant range.

To confirm the emergence of spontaneous spike-and-wave discharges in adult rats (P60+), we performed 48 h of EEG monitoring in animals with a history of P7 hypoxia with or without phenobarbital treatment. Consistent with prior reports (3, 26, 28), recordings showed the emergence of brief electrographic spike-and-wave discharges in animals exposed to hypoxia on P7. Because spike-and-wave discharges are evident at a low level in normal animals across rat strains (44), we compared the seizure number after hypoxia to normoxic controls (mean number of seizures = 32.000; mean total seizure time = 95 s, *n* = 8). The number of seizures and the total electrographic seizure duration during the 48 h recording session was increased in animals with a history of hypoxia (mean number of SWD = 243; mean total SWD time = 802 s, *n* = 10) as compared to normoxic controls (mean number of SWD = 160; mean total SWD time = 536 s, n = 12) (Figures 2F,G; Kruskal-Wallis test). PB did not modify the changes induced by hypoxia. We did not observe longer duration events or behavioral seizures in any animal subjected to chronic monitoring.

We tracked weight gain in a subset of animals: on P7, the day we performed the experimental interventions (hypoxia, drug treatment), and on P13 or P14 or P15 or P29-31 (when animals were euthanized to cut slices). We found no differences in weight between the different treatments (Supplementary Figure S1), aside from a transient decrease in body weight in phenobarbital-exposed male pups in the normoxia group on P13.

### 3.2 Neither hypoxia nor drug treatment modify hippocampal excitability

**Figure 3:**
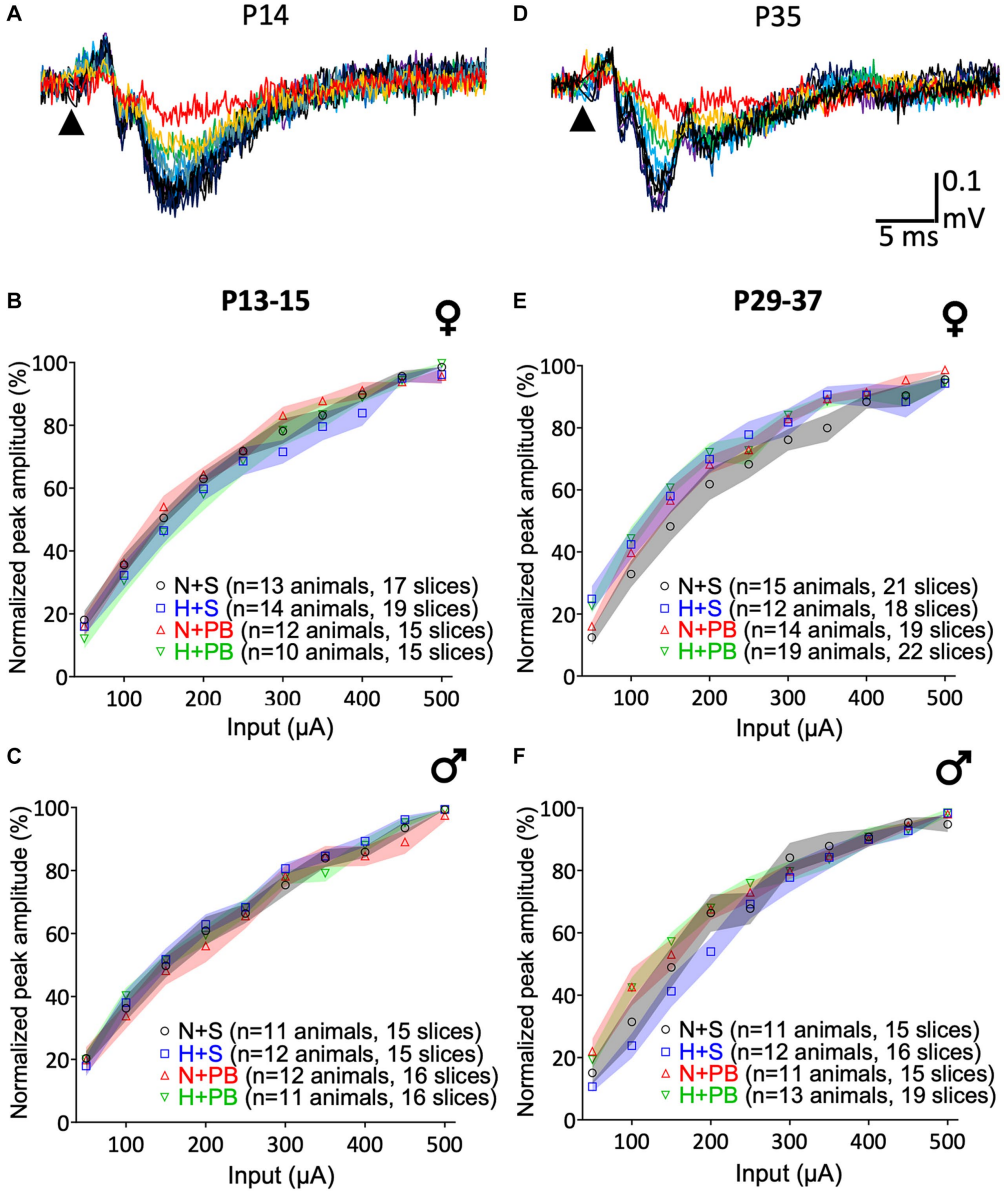
Input/Output (I/O) curves do not differ as a function of history of hypoxia or drug treatment. **(A)** Representative I/O curve from a slice from the P13+ group rat. The arrowhead indicates the time of stimulus pulse delivery. I/O curves of slices from P13+ rats. Females are shown in **(B)**, and males in **(C)**. **(D)** Example of an I/O curve from a slice from the P29+ group rat. The arrowhead indicates the time of stimulus pulse delivery. I/O curves of slices from P29+ rats separated by sex. Females are shown in **(E)** and males in **(F)**. Data were normalized to the maximum response of the I/O curve on a slice-by-slice basis. Colored bands indicate the SEM for each group. The Normoxia-Saline (NS) group is shown in yellow, Hypoxia-Saline (HS) shown in blue, Normoxia-PB (NP) shown in red, and Hypoxia-PB (HP) shown in green. 3-way ANOVA, no post-test. No significant differences between groups.

As shown in Figure 3, all groups, sexes, and ages displayed equivalent input–output curves. As expected, increasing stimulus intensity resulted in larger peak amplitudes of the local field potential response in CA1. Example LFP recordings are shown as superimposed traces in Figures 3A,D, P13+ and P29+ age groups, respectively. These data are quantified in Figures 3B,C for P13+ animals and Figures 3E,F for P29+ animals.

We performed independent three-way ANOVAs for each age and sex with current intensity as a repeated measure and hypoxia and drug treatment as between-subject factors. In all cases, we detected significant main effects of current input – increasing current was associated with increasing response (F_2.2,99_ = 460.2, *p* < 0.0001 for P13+ females, F_2.1,87.0_ = 518.8, *p* < 0.0001 for P13-15 males, F_2.5,141.1_ = 354.5, *p* < 0.0001 for P29+ females, F_2.3,97.3_ = 334.0, *p* < 0.0001 for P29-37 males). We failed to detect main effects of hypoxia, drug treatment, or any interaction between these two variables for any of the groups.

### 3.3 Treatments did not modify post-tetanic potentiation, independently of the induction protocol used

**Figure 4:**
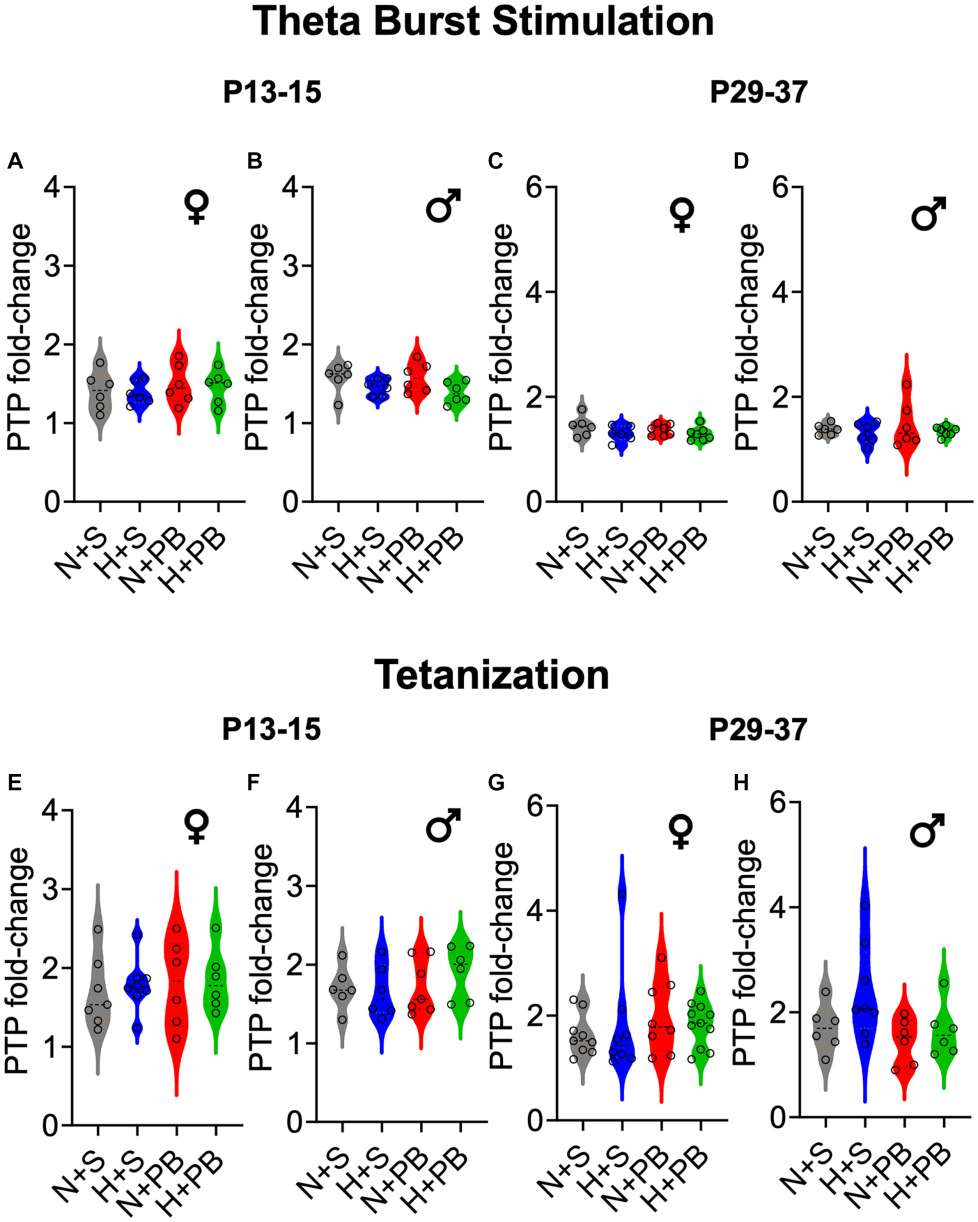
Post-tetanic potentiation (PTP) does not differ as a function of hypoxia history, drug treatment, sex, or age. PTP is shown as fold change over the pre-stimulation baseline. Therefore, values greater than 1 indicate post-tetanic potentiation. Violin plots showing PTP for **(A)** P13+ females, **(B)** P13+ males, **(C)** P29+ females, and **(D)** P29+ males, after the TBS induction protocol. As in **(A–D)** PTP is shown as fold change for the tetanization induction protocol. Violin plots showing PTP for **(E)** P13+ females, **(F)** P13+ males, **(G)** P29+ females, and **(H)** P29+ males. ANOVA revealed no significant differences between any groups. See Figure 5 for group traces illustrating post-tetanic potentiation.

**Figure 5:**
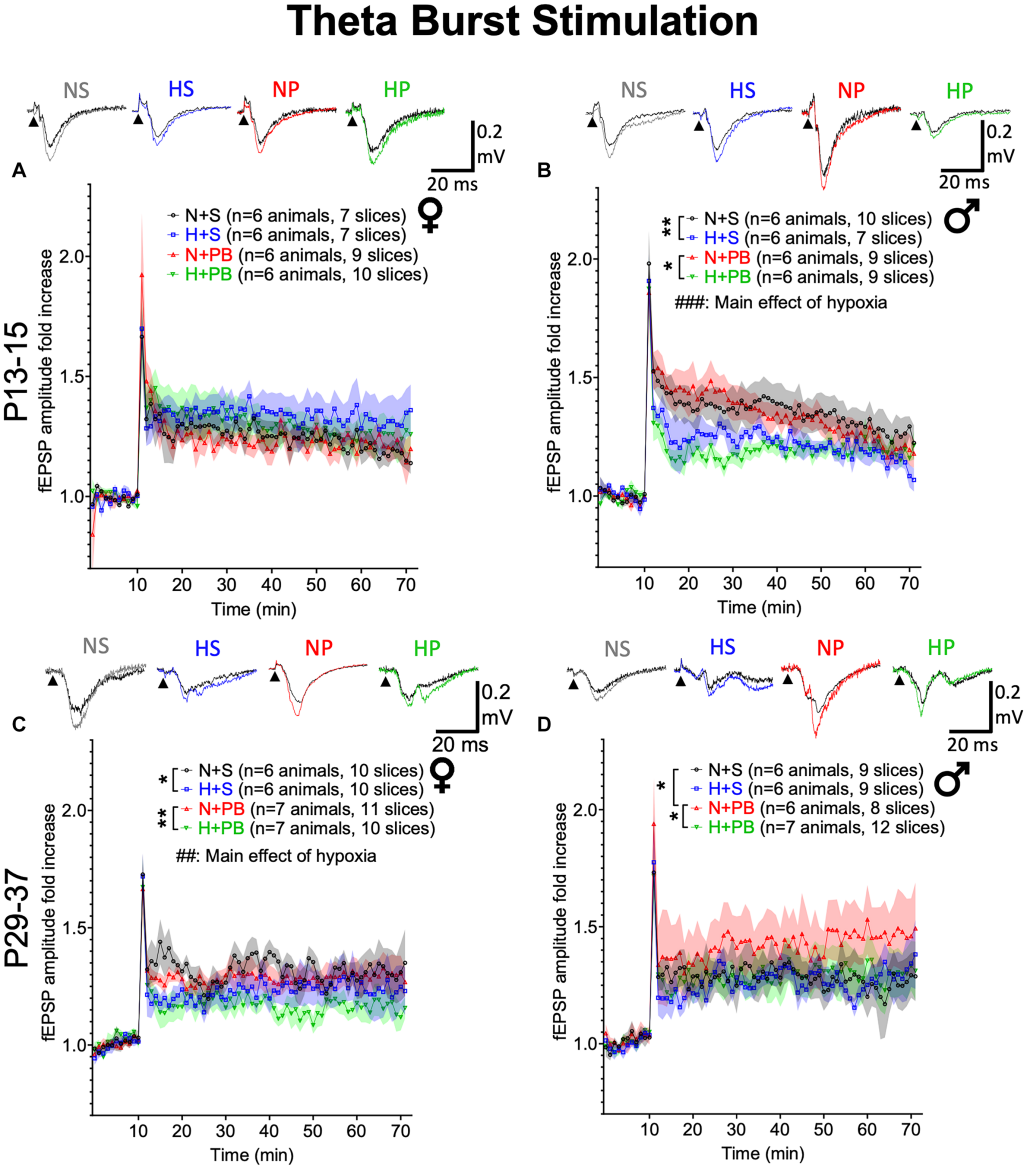
Neonatal hypoxia significantly reduces LTP induced by theta-burst stimulation in a sex-and age-dependent manner. **(A)** Fold change in fEPSP amplitude in slices from P13+ female rats. Inset traces show representative fEPSPs before and after TBS. **(B)** Fold change in fEPSP amplitude in slices from P13+ male rats. **(C)** Fold change in fEPSP amplitude in slices from P29+ female rats. **(D)** Fold change in fEPSP amplitude in slices from P29+ male rats. ## and ### – Significant main effect of hypoxia, *P*s <0.01 and < 0.001, respectively. *,** Significant pairwise comparison between treatment groups, *p* < 0.05 and *p* < 0.01, respectively.

We next analyzed the post-tetanic potentiation (PTP) during the first 5 min after applying the LTP induction protocol to our slices. As shown in Figure 4, treatment groups did not differ in the degree of post-tetanic potentiation (also see average traces in Figure 5). For the TBS (Figures 4A–D) induction protocol, we performed separate two-way ANOVAs for each sex and age and found no significant main effects of drug, hypoxia status, or interaction between these variables (*p* > 0.05 for all comparisons; two-way ANOVA followed by Sidak test). For the tetanizing stimulation protocol (Figures 4E–H), we likewise performed separate two-way ANOVAs for each sex and age and found no significant main effects of drug, hypoxia status, or interaction between these variables (*p* > 0.05 for all comparisons, two-way ANOVA followed by Sidak test).

### 3.4 Under a theta burst stimulation protocol, the treatments modified hippocampal LTP in an age- and sex-dependent manner

To determine if our treatments modified LTP, we next examined LTP induced by TBS (Figure 5). LTP responses did not differ between experimental treatment groups in P13+ females (Figure 5A; Drug: Hypoxia: Interaction). By contrast, in slices from P13+ males (Figure 5B), we found a main effect of hypoxia (F_1,45.491_ = 14.391, *p* < 0.001), driven by a reduction in early-phase LTP in the two hypoxic groups. We did not observe a significant main effect of drug treatment (F_1,45.491_ = 0.418, *p* = 0.521) nor a significant interaction between hypoxia status and drug treatment (F_1,45.491_ = 0.074, *p* = 0.787). Pairwise comparisons revealed significantly higher LTP in the normoxia-saline group compared to the hypoxia-saline group (*p* = 0.006, Sidak). Similarly, the normoxia-PB group displayed significantly higher LTP than the hypoxia-PB group (*p* = 0.016, Sidak).

In P29+ rats (Figures 5C,D), there were no significant effects of drug, hypoxia, nor a drug-by-hypoxia interaction in males. By contrast, in females, we found a significant main effect of hypoxia (F_1,68.334_ = 12.710, *p* = 0.001), but no significant main effect of drug treatment (F_1,68.334_ = 3.324, *p* = 0.073), nor a drug treatment-by-hypoxia interaction (F_1,68.334_ = 0.145, *p* = 0.705). For normoxic males, pairwise comparisons revealed significantly higher LTP in P29+ males treated with PB (*p* = 0.044, Sidak). This PB-mediated increase in LTP was absent when PB was administered after hypoxia (N-PB vs. H-PB: *p* = 0.047, Sidak). Pairwise comparisons revealed that hypoxia reduced LTP for females, independently of their drug treatment: LTP was significantly higher in the normoxia-saline group than the hypoxia-saline group (*p* = 0.033, Sidak) and also in the normoxia-PB group, as compared to hypoxia-PB group (*p* = 0.005, Sidak). Thus, with a TBS-induction protocol, we found age-and sex-dependent impacts of hypoxia and drug treatment on induction of LTP.

### 3.5 Under a tetanization protocol, only hypoxia-exposed P29+ males showed an increase in hippocampal LTP

We next examined LTP using a tetanizing stimulation protocol. The degree of LTP induction differs between TBS and tetanizing stimulation, particularly as a function of age (27, 28). While TBS likely represents a more physiological stimulation protocol, we found that tetanizing stimulation produced stronger LTP in our slices. Furthermore, tetanizing stimulation is a standard LTP induction paradigm (45, 46) and has previously been examined following early-life hypoxia (17).

**Figure 6:**
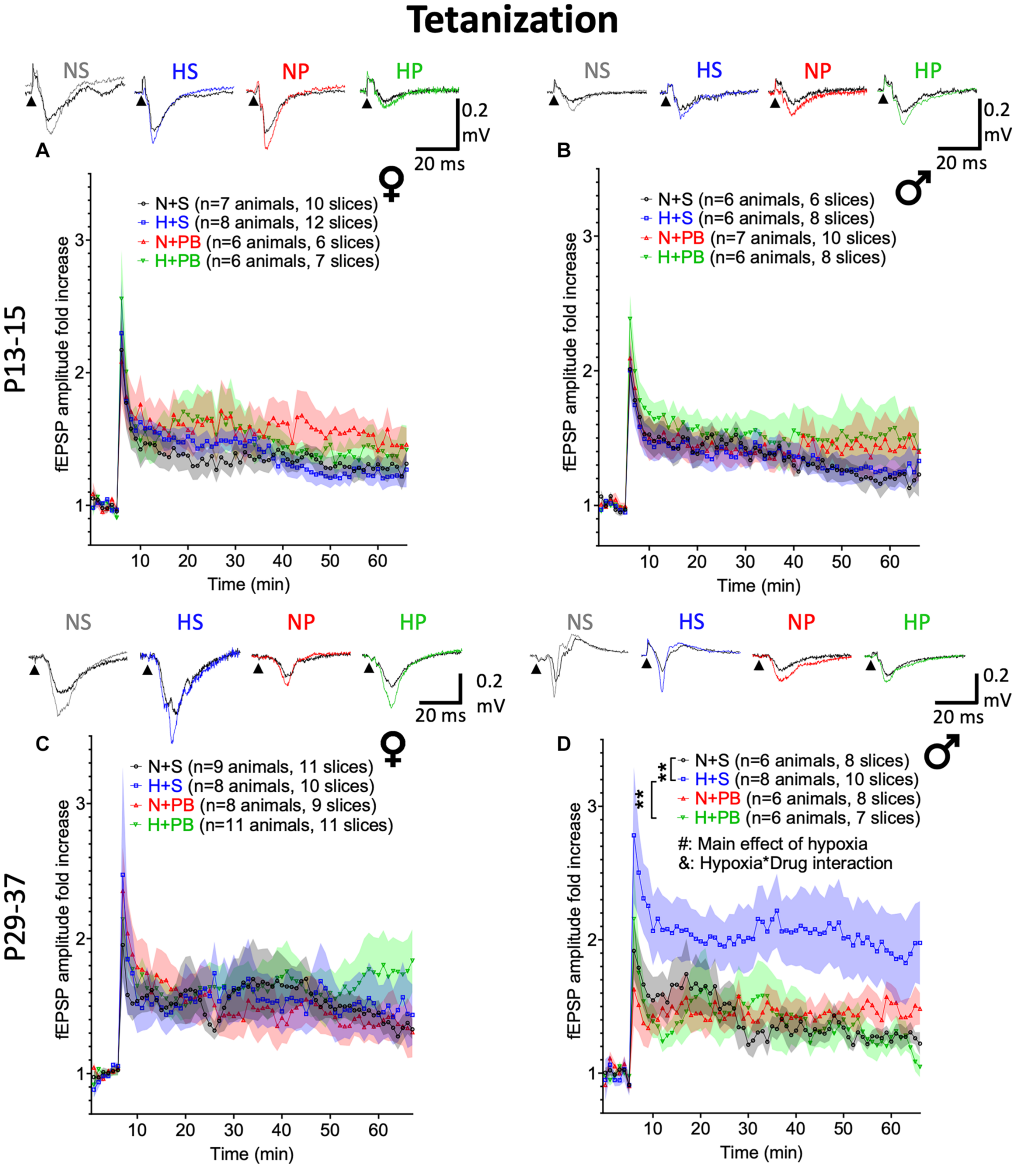
Phenobarbital prevents hypoxia-induced increases in LTP in P29+ males. LTP experiments using a tetanizing stimulation protocol. **(A)** Fold change in fEPSP amplitude in slices from P13+ female rats. Inset traces show representative fEPSPs before and after tetanizing stimulation. **(B)** Fold change in fEPSP amplitude in slices from P13+ male rats. **(C)** Fold change in fEPSP amplitude in slices from P29+ female rats. **(D)** Fold change in fEPSP amplitude in slices from P29+ male rats. # – Significant main effect of hypoxia, *p* < 0.05; & – Significant hypoxia by drug interaction, *p* < 0.05. *,** Significant pairwise comparison between treatment groups, *p* < 0.01.

With the tetanization stimulation protocol, we found no significant effects of hypoxia status, drug treatment, nor any significant interactions in slices from P13+ male or female animals (Figures 6A,B, see Supplementary results for statistics). We also found no significant differences between groups in slices from P29+ females (Figure 6C, see Supplementary results for statistics).

We did, however, detect a robust effect of hypoxia in slices from P29+ males (Figure 6D; F_1,24.298_ = 4.384, *p* = 0.047), a borderline significant main effect of drug treatment (F_1,24.298_ = 4.199, *p* = 0.051), and a significant drug-by-hypoxia interaction (F_1,24.298_ = 4.592, *p* = 0.042). In pairwise comparisons of vehicle-treated males, hypoxia resulted in a significant increase in LTP (*p* = 0.005; Sidak). Interestingly, PB treatment *normalized* the LTP response in hypoxia-exposed animals (hypoxia-saline vs. hypoxia-PB: *p* = 0.005, Sidak) but was without effect in animals that were not exposed to hypoxia (*p* = 0.949, Sidak).

## 4 Discussion

In this study, we describe the individual and combined effects of a single episode of hypoxia and a single exposure to PB on the development of LTP at the Schaeffer collateral-CA1 synapse in P7 rat pups. We found that hypoxia, PB, or the combination of these factors produced age-and sex-specific effects, which differ as a function of the protocol used to induce LTP. The combination of PB and hypoxia normalized changes in LTP induced by hypoxia in the tetanization protocol and normalized changes induced by PB in the TBS protocol. Thus, PB-treated animals with a history of hypoxia showed normal LTP, which suggest that there is *no* additive toxicity associated with PB treatment on the background of hypoxia, at least with respect to LTP. We performed our experiments at time points well after a single dose PB is expected to be cleared, to separate out acute impacts on excitability from lasting changes in brain function.

Prior studies have suggested that neonatal hypoxia (17) and PB exposure (10) can induce long-term changes in excitatory/inhibitory balance within the hippocampus. We found no differences in the input–output response as a function of either hypoxia or drug treatment, suggesting no change in the excitability of the circuit. Similarly, these treatments did not affect post-tetanic potentiation (PTP), a form of presynaptic plasticity. The lack of differences in the I/O and PTP responses suggests that the differences in LTP we observe are not due to impaired induction but rather LTP maintenance.

As expected on the basis of prior studies, neonatal hypoxia was associated with a substantial spike-and-wave discharge burden later in life. Interestingly, while PB normalized LTP defects caused by neonatal hypoxia, it did not modify the spike-and-wave discharge burden. This may reflect different processes, or different circuit engagement, as the spike-and-wave discharges likely have preponderant thalamic involvement although they have also been observed in hippocampus (44).

We observed different extents of LTP between the TBS and the tetanization protocols. We used theta burst stimulation to maximize LTP in juvenile rats (45) and tetanization, which is the most frequently used protocol to analyze hypoxic seizure-induced changes in LTP (15–17, 47). These two protocols have previously resulted in different outcomes in the same hippocampal preparation (48, 49). Some studies have suggested molecular mechanisms that differ between the two induction methods. BDNF signaling through TrKB receptors is necessary for the induction of LTP by TBS but not by tetanization (50, 51). By contrast, tetanization-induced LTP, but not TBS-induced LTP, depends on metabotropic glutamate receptor signaling (52). While we did not examine the mechanism(s) underlying these differences in the present study, further examination of the BDNF/TrkB and mGluR pathways after hypoxia and drug exposure may provide an informative area for future exploration.

The induction protocols we used revealed sex differences in LTP impairment: the TBS protocol showed an impairment in LTP caused by hypoxia that affected males at P13+ and females at P29+. The TBS protocol also showed an increase in LTP on P29+ males only. Estrogens and progesterone are neuroprotective agents that contribute to the higher survival rate, lower morbidity, and reduced neuronal apoptosis of females after brain injury (53). Accordingly, some have suggested that female rodents would have better cognitive outcomes than males after neonatal hypoxia (23). By contrast, females display enhanced sensitivity to hypoxia-induced apoptosis than males (21), and females have more severe memory deficits after neonatal hypoxia-ischemia (22). No studies have compared the impact of neonatal PB in females and males. However, the shift from depolarizing-to-hyperpolarizing GABA occurs later in male rats than in female rats (54). This may contribute to the higher vulnerability of male rats to PB treatment. In the male hippocampus, PB would have an excitatory effect at P7, while in females, its effect would be mostly inhibitory. This may underlie some of the sex-dependent effects we observed. Several studies have shown that LTP is easier to induce in prepubertal females than males and puberty reverses this trend (55). Population field responses in hippocampus, AMPA/kainate mediated currents, and the magnitude of LTP are positively modulated by estrogen levels, and both baseline synaptic transmission and LTP are negatively modulated by progesterone (56). Moreover, sex differences have been reported in the sources of calcium required for LTP induction by estradiol (with females requiring both L-type calcium channel activation and release of calcium from intracellular stores, whereas in males either source of calcium is sufficient) (57). These studies indicate that LTP is influenced by sex, and therefore may explain the sex differences we observed while also highlighting the need for conducting LTP studies that include both sexes.

In slices from P29+ male rats exposed to neonatal graded hypoxia protocol and using the tetanization induction protocol, we found enhanced LTP. While this is consistent with one of the first studies of neonatal hypoxia from the Jensen group (16), they did not evaluate time points beyond the acute post-insult period. This result – and our present findings – differ from previous studies reporting impairment in hippocampal LTP in slices both in the neonatal period and later in life, as well as in slices (17, 58) and *in vivo* (47), although one study found an increase in postsynaptic calcium levels-which is associated with NMDA-dependent LTP-after neonatal graded hypoxia (15). Several differences between our study and others might explain the different outcomes. First, ours is the first study to evaluate sex differences in this context; prior studies only examined males. Second, we perform the graded hypoxia protocol at P7, while previous studies did so at P10 (15–17, 26, 28, 59). The developmental switch from excitatory to inhibitory action of GABA in the hippocampus occurs between P5 and P7 (60), meaning that, at P7, this shift may not be complete. The range of P7 to P10 corresponds approximately to a stage equivalent to full term in humans, depending on the precise developmental milestone (61, 62). We selected P7 because it represents the peak of the brain growth spurt, a period during which there is highest vulnerability to drug-induced neurotoxicity. Both term- and pre-term hypoxia are relevant clinical conditions, and in early development, small differences in timing can lead to large changes in outcomes.

While the graded global hypoxia model has been widely used, and clearly models some of the salient features of HIE (e.g., acute and subchronic seizures, long term risk of epileptogenesis, cognitive disruption) (3, 28, 59), it does not include an ischemic component. Indeed, models of hypoxia-ischemia, which rely unilateral ligation of the common carotid, produce a more severe insult than the graded hypoxia or other hypoxia models (19). It is possible that the impact of PB would differ in the carotid ligation model, however, this is complicated by the need for anesthesia, which has *per se* effects on brain development (27). This was a critical consideration for our selection of the graded global hypoxia model in the present study, as our primary goal was to determine the impact and interaction between early-life seizures and PB.

PB exposure during the neonatal period increases neurodegeneration, impairs neurogenesis, and disrupts synaptic development (7–10, 12–14). However, the overwhelming majority of these studies (including those from our lab) examined the impact of PB exposure in otherwise normal animals. While this is relevant to *in utero* exposure during the third trimester, it is an incomplete model of the neonatal condition. Neurologically intact infants are not given PB. Our present study joins only a small number of preclinical studies in considering the impact of PB on the background of epileptic seizures: (1) Quinlan et al. (20) used a hypoxia-induced seizure model in mice. PB injection did not worsen the behavioral sequelae of the seizures compared to the animals that received hypoxia alone. This seems to indicate that PB did not prevent the deficits caused by hypoxia, but it did not add to them either. (2) The combination of PB and hypothermia after a hypoxic–ischemic (not purely hypoxic) insult reduced brain volume loss and improved motor function significantly, compared to hypothermia alone (63). (3) However, another study of carotid ligation found normalization of weight gain and exploratory behavior with a lower (30 mg/kg) but not a higher dose (60 mg/kg) of PB (40). This is particularly relevant to the current study, where we selected a higher dose of PB. (4) Finally, Torolira et al. (64) found that PB did not exacerbate status epilepticus-induced neurodegeneration in the hippocampus, but did exacerbate it in other regions (e.g., the cortex, basal ganglia). Clinical studies have also explored the neuroprotective effects of PB, with varied results: A small prospective, randomized clinical trial showed that 40 mg/kg of PB given to term infants with perinatal asphyxia did not reduce the number of infants with seizures, but did improve long term outcomes (65). Together, preclinical and clinical studies paint a complicated picture regarding the interaction of PB and hypoxia/asphyxia/HIE. Future studies directly examining the interaction between global graded hypoxia and PB on behavioral outcomes are needed to address the degree to which LTP deficits translate (or not) into learning and memory dysfunction.

While we used a relatively high dose of PB in the present study, we also note that a typical intravenous loading dose of 20 mg/kg PB in a human infant is not equivalent to a 20 mg/kg administered IP in a neonatal rat. IP administration is typically associated with reduced – and often much more variable – bioavailability. Accordingly, the dose we selected (75 mg/kg) is at the high end of the therapeutically relevant range in animal models (29–32), and produces plasma concentrations that exceed typical starting doses but are likely relevant for cases of refractory seizures (41–43). In addition to the above reasons, we selected this dose in part based on the robust induction of neuronal apoptosis it causes in P7 animals (8), and in part because it produces clear alterations in synaptic development in the hippocampus and striatum (9, 10). From a toxicological perspective, a benign profile at the high end of the therapeutically relevant range is an important safety signal. This is precisely what we observed. The combination of high dose PB with neonatal hypoxia did not worsen – and in some cases ameliorated – hypoxia-provoked alterations in LTP. Our present studies underscore the importance of considering drug toxicity in the context of seizure history.

## Data availability statement

The raw data supporting the conclusions of this article will be made available by the authors, without undue reservation.

## Ethics statement

The animal study was approved by the Georgetown University Institutional Animal Care and Use Committee (Protocol #2016–1306) in accordance with the Guide for the Care and Use of Laboratory Animals. The study was conducted in accordance with the local legislation and institutional requirements.

## Author contributions

IS-B: Data curation, Formal analysis, Investigation, Methodology, Project administration, Visualization, Writing – review & editing, Writing – original draft. AG: Investigation, Writing – review & editing. VG: Investigation, Writing – review & editing. SQ: Investigation, Writing – review & editing. EW: Investigation, Writing – review & editing. SV: Investigation, Writing – review & editing, Conceptualization, Data curation, Formal analysis, Methodology, Supervision. PF: Investigation, Writing – review & editing, Conceptualization, Data curation, Formal analysis, Funding acquisition, Methodology, Project administration, Supervision, Visualization.
