# Supplementary material for: Phenobarbital does not worsen outcomes of neonatal hypoxia on hippocampal LTP on rats
Source: Front Neurol. 2023 Nov 21;14:1295934. doi: 10.3389/fneur.2023.1295934 (PMC10703306; doi:10.3389/fneur.2023.1295934)
Supplement: Supplementary file 1 [file Data_Sheet_1.docx]

Supplementary Material

- 1. **Supplementary Results**

We tracked weight gain in a subset of animals: on P7, the day we performed the experimental interventions (hypoxia, drug treatment), and on P13 or P14 or P15 or P29-31 (when animals were euthanized to cut slices). We found no differences in weight between the different treatments (Supplementary Figure 1), aside from a transient decrease in body weight in phenobarbital exposed male pups in the normoxia group on P13.

**Supplementary Statistical Results for Figure 5.**

We found no significant effect of hypoxia (F_1,34.711_=0.619, P=0.437), drug treatment (F_1,34.711_=2.969, P=0.094) or drug-by-hypoxia interaction (F_1,34.711_=0.355, P=0.555) in slices from P13+ males. Similarly, we found no significant effect of hypoxia (F_1,33.938_=0.067, P=0.798), drug treatment (F_1,33.938_=3.592, P=0.067) or drug-by-hypoxia interaction (F_1,33.938_=0.026, P=0.874) in slices from P13+ females. We also found no significant differences between groups in slices from P29+ females (hypoxia: F_1,30.826_=0.816, P=0.373, drug treatment: F_1,30.826_=0.185, P=0.671, drug-by-hypoxia interaction: F_1,30.826_=0.004, P=0.951).

# Supplementary Figures and Tables

## Supplementary Figures


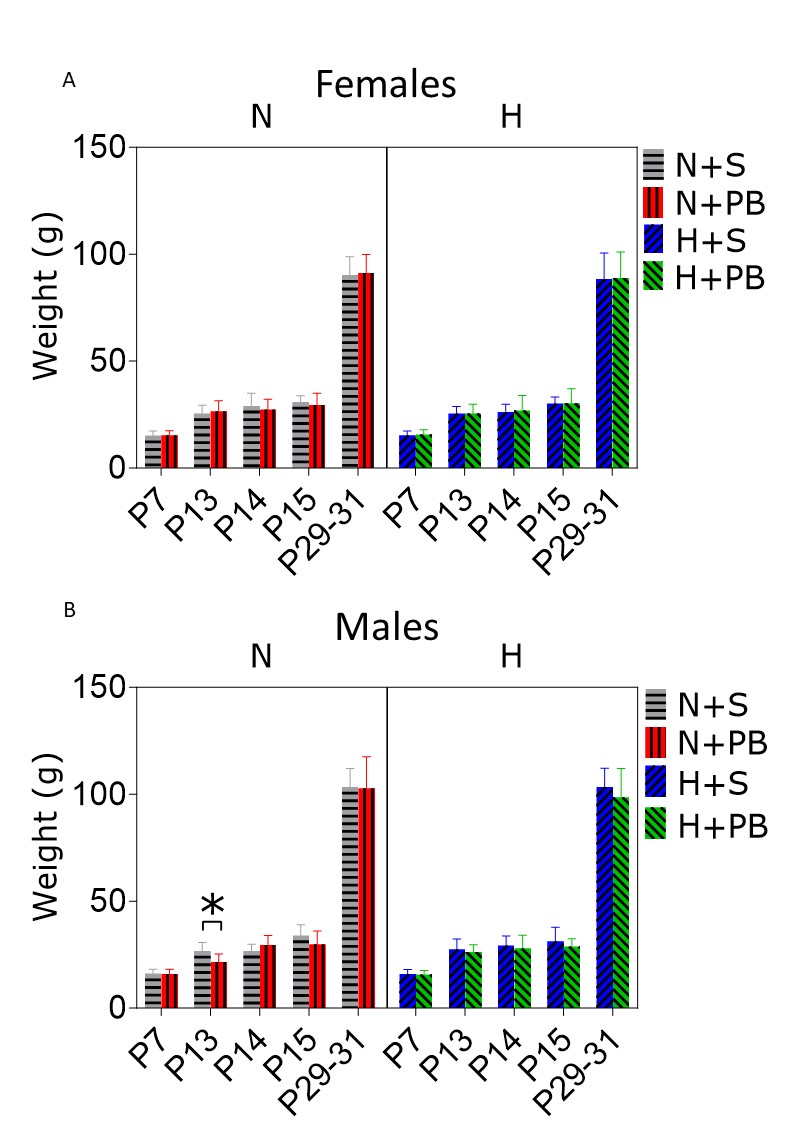


**Supplementary Figure 1.** Phenobarbital injection alone – but not in combination with hypoxia - transiently reduces weight on P13 male rats. A) Weight of female pups at ages between P7 and P29-31. B) Weight of male pups at ages between P7 and P29-31. * = significant differences between group means, P<0.05. Mixed-effects model (REML). Dunnett’s Multiple comparison’s test pairwise comparison between each group mean (H+S, N+PB, H+PB) to the control group mean (N+S).

**Supplementary Tables:**

**Table 1:** Number of animals and litters included in the study for each treatment group and sex

|  | **Male** | | **Female** | |
| --- | --- | --- | --- | --- |
|  | **P13-15** | **P29-37** | **P13-15** | **P29-37** |
| **NS** | 12 (12) | 12 (11) | 13 (12) | 15 (12) |
| **NP** | 13 (11) | 12 (10) | 12 (9) | 15 (13) |
| **HS** | 12 (12) | 14 (11) | 14 (14) | 14 (14) |
| **HP** | 12 (10) | 13 (11) | 12 (9) | 18 (14) |

**Table 2.** Mean and median ages of P29-37 females from TBS experiments.

| **Group** | **Mean age (days)** | **Median age (days)** | **Significant difference among means?**  **P value** |
| --- | --- | --- | --- |
| **NS** | 32.00 | 31.00 | No  P=0.9715 |
| **HS** | 31.50 | 32.00 |  |
| **NP** | 31.71 | 31.00 |  |
| **HP** | 32.00 | 31.00 |  |

**Table 3.** Mean and median ages of P29-37 males from TBS experiments.

| **Group** | **Mean age (days)** | **Median age (days)** | **Significant difference among means?**  **P value** |
| --- | --- | --- | --- |
| **NS** | 30.83 | 30.50 | No  P=0.1664 |
| **HS** | 32.33 | 31.50 |  |
| **NP** | 32.67 | 32.00 |  |
| **HP** | 34.14 | 36.00 |  |

**Table 4.** Mean and median ages of P29-37 females from tetanization experiments.

| **Group** | **Mean age (days)** | **Median age (days)** | **Significant difference among means?**  **P value** |
| --- | --- | --- | --- |
| **NS** | 35.00 | 35.00 | No  P=0.1912 |
| **HS** | 33.63 | 34.00 |  |
| **NP** | 32.38 | 32.50 |  |
| **HP** | 32.00 | 31.00 |  |

**Table 5.** Mean and median ages of P29-37 males from tetanization experiments.

| **Group** | **Mean age (days)** | **Median age (days)** | **Significant difference among means?**  **P value** |
| --- | --- | --- | --- |
| **NS** | 35.50 | 36.00 | No  P=0.4585 |
| **HS** | 33.50 | 33.50 |  |
| **NP** | 32.17 | 32.50 |  |
| **HP** | 34.00 | 35.00 |  |
